# The Impact of Mediterranean Dietary Intervention on Metabolic and Hormonal Parameters According to *BRCA1/2* Variant Type

**DOI:** 10.3389/fgene.2022.820878

**Published:** 2022-03-09

**Authors:** Andreina Oliverio, Paolo Radice, Mara Colombo, Angelo Paradiso, Stefania Tommasi, Antonella Daniele, Daniela Andreina Terribile, Stefano Magno, Donatella Guarino, Siranoush Manoukian, Bernard Peissel, Eleonora Bruno, Patrizia Pasanisi

**Affiliations:** ^1^ Department of Research, Fondazione IRCCS Istituto Nazionale dei Tumori di Milano, Milan, Italy; ^2^ Unit of Molecular Bases of Genetic Risk and Genetic Testing, Fondazione IRCCS Istituto Nazionale dei Tumori di Milano, Milan, Italy; ^3^ Experimental Oncology, Center for Study of Heredo-Familial Tumors, IRCCS Istituto Tumori “Giovanni Paolo II” Bari, Bari, Italy; ^4^ Molecular Diagnostics and Pharmacogenetics Unit, IRCCS Istituto Tumori “Giovanni Paolo II” Bari, Bari, Italy; ^5^ Department of Women Health Area, Università Cattolica S. Cuore, Rome, Italy; ^6^ Department of Women and Child Health and Public Health, Fondazione Policlinico Universitario A. Gemelli IRCCS, Rome, Italy; ^7^ Unit of Medical Genetics, Fondazione IRCCS Istituto Nazionale dei Tumori di Milano, Milan, Italy

**Keywords:** *BRCA1/2* genes, pathogenic variants, insulin, dietary intervention, metabolism

## Abstract

The female carriers of *BRCA1/2* pathogenic variants (mutations) face a high lifetime risk of developing breast and/or ovarian cancer. However, the risk may differ depending on various genetic and non-genetic elements, including metabolic and hormonal factors. We previously showed that a 6-month Mediterranean dietary intervention trial reduced body weight and the levels of insulin-like growth factor I and other metabolic factors in BRCA mutation carriers. We also found that higher baseline levels of glucose and insulin were significantly associated with BRCA loss-of-function (LOF) variants. In this study, we evaluated whether the BRCA mutation type influences in a different way the metabolic and hormonal response to the dietary intervention in 366 female carriers. The LOF variant carriers randomized in the intervention group (IG) showed significantly higher changes in most considered parameters compared to the control group (CG). The nonsynonymous variant carriers in the IG showed similar changes, but none of them were statistically significant. Performing the “delta” analysis of differences (intention-to-treat analysis), we observed that in LOF variant carriers, the reduction of insulin levels was significantly more pronounced that in nonsynonymous variant carriers. These findings suggest that the changes in insulin levels might be modulated by a different response to the dietary intervention mediated by BRCA LOF variants.

## Introduction

The women carriers of pathogenic variants (mutations) in the *BRCA1* and *BRCA2* genes (*BRCA1/2*) show a very high risk of breast cancer (BC) and/or ovarian cancer (OC) (Kuchenbaecker et al., 2017). However, it has been shown that in these individuals, the lifetime risk of BC/OC significantly differs on the basis of the location of the mutations within the genes (Rebbeck et al., 2015) and the presence of the modifying genetic factors that are combined into a polygenic risk score (Barnes et al., 2020). In addition, the occurrence of conditions such as obesity and metabolic syndrome (MS) may increase BC risk in the carriers of BRCA gene mutations (Nkondjock and Ghadirian, 2004; Bissonauth et al., 2009; Pettapiece-Phillips et al., 2015; Dumais et al., 2017). In a previous randomized controlled trial (NCT03066856), we showed that a 6-month dietary intervention based on the Mediterranean diet with moderate protein restriction (to ∼11% of total calorie intake), significantly modifies the potential metabolic-related modulators of BRCA mutation penetrance, such as body weight, insulin-like growth factor I (IGF-I), and MS parameters in the female carriers of the BRCA mutations of the intervention group (IG) compared to the control group (CG) (Pasanisi et al., 2014; Bruno et al., 2018; Pasanisi et al., 2018; Bruno et al., 2020; Daniele et al., 2020; Bruno et al., 2021). In the trial on the participants with a complete genetic test, we also analyzed the association of the baseline metabolic and hormonal factors (before starting the dietary intervention) with the *BRCA1/2* variant type, which are categorized into loss-of- function (LOF) or nonsynonymous variants. This explorative analysis suggested that higher levels of glucose and insulin were significantly associated with BRCA LOF variants (*p* = 0.03 and *p* < 0.001, respectively) (Oliverio et al., 2020). This association seemed stronger in the *BRCA2* LOF variant carriers who, in addition, showed at the baseline a worse metabolic condition compared to nonsynonymous variant carriers (Oliverio et al., 2020). The aim of the present study was to evaluate whether the BRCA pathogenic variant type (LOF vs. nonsynonymous) influences in a different way the metabolic and hormonal responses after a 6-month dietary intervention, i.e., the different changes in factors including insulin and IGF-I levels, body weight, and MS parameters. These changes were further analyzed on the basis of group randomization (IG-nonsynonymous vs. CG-nonsynonymous and IG-LOF vs. CG-LOF). The study included 366 female BRCA mutation carriers who completed our dietary randomized controlled trial.

## Patients and Methods

### Study Subjects

Detailed information regarding the Italian multicenter, prospective, dietary randomized controlled trial (NCT03066856) and its main results have already been reported (Pasanisi et al., 2014; Bruno et al., 2018; Pasanisi et al., 2018; Bruno et al., 2020; Daniele et al., 2020; Oliverio et al., 2020; Bruno et al., 2021). Following the inclusion criteria of the above trial, study subjects were recruited through family clinics and patient associations among women (18–70 years) carriers of *BRCA1/2* mutations, with or without a previous diagnosis of BC/OC and without a clinical evidence of metastases. Unaffected women who underwent bilateral prophylactic mastectomy were excluded from the cohort. All participants signed informed consent for participating into the study and received general recommendations for the prevention of cancer (the World Cancer Research Fund /AICR decalogue) (World Cancer Research Fund and American Institute for Cancer Research, 2007). After the baseline examinations, women were randomly assigned to an active dietary intervention group (IG) or to a control group (CG). The center, age (≤40 and >40 years), and biological relationship were considered to balance the randomization. The study was approved by the Ethics Committee of the coordinating center, the Fondazione IRCCS Istituto Nazionale dei Tumori (INT) di Milano, Italy (approval number: INT106/13).

### Data Collection and Measurements

Women were asked to provide information on their *BRCA1/2* mutational status and, whenever possible, the complete report of the genetic test and to fill in a questionnaire on their medical history and major cancer risk factors, including menstrual and reproductive history and behavioral factors. According to the trial protocol (Pasanisi et al., 2014), at the baseline and at the end of the 6-month dietary intervention, the women were requested to attend anthropometric and body composition measurements, to provide a 20 ml blood sample for metabolic and hormonal assays, and to complete a 24 h food frequency diary of the previous day and the validated 14-point MEDAS questionnaire (Schröder et al., 2011).

### Dietary Intervention

The dietary intervention has been fully described elsewhere (Pasanisi et al., 2014; Bruno et al., 2020; Bruno et al., 2021). Briefly, the women randomized into the IG attended six full days of activities over the following 6 months. These activities included six kitchen courses with subsequent lunch and six nutritional conferences. The women were recommended to reduce calorie intake and animal food in order to lower protein intake to ∼11% of the total calorie intake (mainly reducing milk and animal protein), high-glycemic-index food, and the sources of saturated fat, and to eat mostly food of plant origin. The women learned how to prepare traditional Mediterranean dishes based on whole-grain cereals (grains/pasta) and legumes, seasoned with vegetable sauces and little fat, and cakes and cookies without sugar, milk, butter, and refined flour, using instead dried fruit, such as raisins and apricots, oleaginous seeds, soy milk, cereal flakes, or unrefined flour.

### Laboratory Methods

The collected blood samples were stored at −80°C. Plasma glucose, triglycerides, and total low-density lipoprotein and high-density lipoprotein cholesterol, were measured using routine clinical laboratory techniques. Serum IGF-I (Biosource, Nivelles, Belgium) and insulin levels (Immunotech, Prague, Czech Republic) were measured using commercial kits as described (Bruno et al., 2020). All tests were performed blinded to the participant’s disease status.

### Nomenclature and Classification of Variants

Detailed information regarding the variant nomenclature and classification has been previously described (Oliverio et al., 2020). The pathogenic or likely pathogenic variants were divided into two groups, according to their predicted effect:

• LOF variants, including frameshift, nonsense, large deletion, and spliceogenic variants, introducing, or predicted to introduce, a premature termination codon (PTC) or leading to the in-frame loss of gene regions coding for functional domains
• Nonsynonymous variants, including missense and small in-frame deletion (Supplementary Table S1)

### Statistical Analysis

The general metabolic and dietary characteristics were summarized by pathogenic variants using means and standard deviation (SD) or frequencies, and they were compared using t-tests or χ^2^. The body mass index (BMI) was defined as body weight/squared height (kg/m^2^). The data about dietary consumption were collected from the 24 h food frequency diaries about the previous day’s intake of single food items and food groups. Food group variables were generated by summing single food items: sugary food and beverages (white sugar + brown sugar + malt + chocolate + candies + sugary beverages); refined grain products (white bread + white rice + egg noodles + biscuits + corn flakes + sweetened muesli); whole-grain products (whole bread + whole rice + other whole grain cereals + unsweetened muesli + oat flakes); legumes and soy products (legumes + tofu/tempeh); dairy products (milk + other dairy products); red/processed meat (red meat + processed meat); total animal products (white meat + eggs + fish + dairy + red meat + processed meat); and alcoholic beverages (wine + beer + spirits). A two-sample *t*-test was used to compare baseline and 6-month measurements (before–after analysis) by group randomization and by the two pathogenic variant groups.

ANOVA was used to analyze the differences (*delta, Δ*) between the end of the study and the baseline values of metabolic parameters/food frequency consumptions in nonsynonymous and LOF carriers by the two randomization groups (IG vs. CG, intention-to-treat analysis). We also assessed the “delta” analysis of differences including the pathogenic variant type into the model and controlling for the study center, age (tertiles), BMI (tertiles), and the baseline value of the variables under study.

A *p*-value of <0.05 was taken as significant. All statistical tests were two sided. Analyses were done using the STATA 16 statistical package (StataCorp, College Station, TX, United States).

## Results

Out of 502 women who joined our dietary intervention trial, 438 had a complete genetic test. Among them, 375 (85.6%) were ascertained as the carriers of LOF variants and 63 (14.4%) of nonsynonymous variants in *BRCA1/2* genes. The general characteristics of the study population are reported in Table 1. Among nonsynonymous variant carriers, those with *BRCA1* mutations (71.4%) were more represented than in LOF variant carriers (59.7%) (*p* = 0.08). The history of pregnancy slightly differed between LOF and nonsynonymous variant carriers, with a younger age at first live birth (*p* = 0.07) and a higher number of children in the former group (*p* = 0.41). The LOF variant carriers showed a slightly lower level of education and higher frequency of natural menopause compared to nonsynonymous variant carriers. As for the disease status, 216 (82.4%) women had a previous diagnosis of BC, 10 (3.8%) had both BC and OC, 36 (13.7%) had OC, and 176 were unaffected. Among the affected women, 228 had LOF variants and 34 had nonsynonymous variants. Among BC cases, the women with nonsynonymous variants were significantly more affected by estrogen receptor-negative tumors (*p* = 0.03). Concerning food frequency consumption, at the baseline, the two groups were fairly homogeneous as regards the consumption of recommended and not-recommended food. However, nonsynonymous variant carriers showed a slightly higher consumption of vegetables (*p* = 0.06) and legumes/soy products (*p* = 0.08), while LOF variant carriers showed a slightly higher consumption of sugary foods (*p* = 0.35) (data not shown).

**TABLE 1:** Baseline characteristics of the study population by pathogenic variant type.

Variables	Total population (438)	Nonsynoymous (63)	LOF (375)	*p*-value^*^
Age (years)	46.9 ± 11.1	46.5 ± 10.7	47.0 ± 11.2	0.75
Gene mutation (%)				
*BRCA1*	61.4	71.4	59.7	
*BRCA2*	38.6	28.6	40.3	0.08
Education (%)				
First level	16.7	15.9	16.8	
Second level	44.5	38.1	45.6	
Third level	38.8	46.0	37.6	0.08
Menarche (years)	12.4 ± 1.4	12.3 ± 1.4	12.4 ± 1.5	0.88
Age at first live birth (years)	29.0 ± 4.9	30.2 ± 5.5	28.7 ± 4.8	0.07
Pregnancy (yes) (%)	71.0	71.0	71.1	0.99
Number of children (%)				
1	30.3	35.7	29.4	
≥2	69.7	64.3	70.6	0.41
Menopause (%)	74.7	69.8	74.5	0.34
Natural menopause (%)	24.0	16.3	25.2	0.21
Oral contraceptive use in the past (%)	67.8	72.6	67.0	0.39
Smoked in the past (%)	27.3	32.3	26.5	0.30
	**Total affected (262)**	**Nonsynonymous (34)**	**LOF (228)**	
Cancer type (% if affected)				
Breast	82.4	88.2	81.6	
Breast and ovarian	3.8	5.9	3.5	
Ovarian	13.7	5.9	14.9	0.31
Age at diagnosis (years)	43.1 ± 10.0	42.4 ± 9.1	43.2 ± 10.1	0.56
	**Breast cancer (216)**	**Nonsynonymous (30)**	**LOF (186)**	
Infiltrating duct (%)	85.3	93.8	83.8	0.87
ER-negative (%)	**50.7**	**71.9**	**47.0**	**0.03**
Axillary node metastasis (%)	20.9	17.9	21.4	0.13
Ki-67 > 14 (%)	95.4	94.4	95.5	0.73

* p of *t*-test or χ^2^ as appropriate.

Overall, 366 women, including 193 randomized into the IG and 173 in the CG, concluded the 6-month dietary intervention and were available for the final examinations. Among these, 312 were carriers of LOF variants and 54 of nonsynonymous variants. At the end of the 6-month intervention, all women significantly improved most of the parameters under study, with a major effect in the IG (Supplementary Table S2).

Table 2 shows the before–after analysis in IG and CG by the pathogenic variant type. LOF variant carriers in the IG experienced a higher improvement of the majority of the parameters under study, while nonsynonymous carriers in the IG showed a significant reduction only in weight, BMI, waist circumferences, glycemia, and total cholesterol levels. As concerned women were randomized into the CG, only LOF variant carriers showed significant improvements of anthropometric and metabolic parameters (Table 2).

**TABLE 2:** Results of before–after analysis by pathogenic variant type.

	Nonsynonymous CG (*N* = 16)			Nonsynonymous IG (*N* = 38)			LOF CG (*N* = 157)			LOF IG (*N* = 155)		
	Baseline Mean ± SD	Six months Mean ± SD	*p* *	Baseline Mean ± SD	Six months Mean ± SD	*p* *	Baseline Mean ± SD	Six months Mean ± SD	*p* *	Baseline Mean ± SD	Six months Mean ± SD	*p* *
Weight (kg)	67.0 ± 15.4	66.3 ± 13.8	0.44	**64.5 ± 10.8**	**63.2 ± 11.4**	**0.03**	**65.2 ± 13.6**	**64.5 ± 13.5**	**<0.01**	**61.5 ± 10.5**	**60.1 ± 10.5**	**<0.01**
BMI (kg/m^2^)	25.1 ± 6.1	24.9 ± 5.5	0.43	**25.1 ± 4.5**	**24.5 ± 4.5**	**0.03**	**24.7 ± 4.8**	**24.4 ± 4.7**	**<0.01**	**23.6 ± 4.3**	**23.1 ± 4.2**	**<0.01**
Waist circumference (cm)	78.3 ± 12.9	78.0 ± 12.3	0.74	**79.3 ± 10.9**	**77.2 ± 12.0**	**0.04**	**79.0 ± 13.3**	**78.2 ± 12.6**	**0.03**	**76.4 ± 10.4**	**74.6 ± 10.0**	**<0.01**
Hip circumference (cm)	104.7 ± 11.1	104.2 ± 9.8	0.53	100.5 ± 7.6	99.3 ± 8.7	0.06	100.4 ± 10.0	99.9 ± 10.2	0.17	**97.9 ± 9.3**	**96.2 ± 8.8**	**<0.01**
Systolic pressure (mmHg)	119.8 ± 16.8	123.1 ± 14.7	0.27	124.3 ± 15.5	124.6 ± 12.8	0.91	**124.9 ± 15.4**	**120.8 ± 13.8**	**<0.01**	**126.6 ± 18.6**	**122.5 ± 15.3**	**<0.01**
Diastolic pressure (mmHg)	80.3 ± 10.5	80.4 ± 9.1	0.91	83.2 ± 11.6	81.9 ± 10.5	0.46	**81.4 ± 10.6**	**78.9 ± 9.1**	**<0.01**	**82.1 ± 11.1**	**79.4 ± 11.0**	**<0.01**
Glycemia (mg/dl)	100.7 ± 22.3	89.9 ± 21.8	0.18	**98.4 ± 21.9**	**90.2 ± 14.6**	**0.03**	**102.3 ± 24.4**	**93.4 ± 20.0**	**<0.01**	**101.7 ± 22.3**	**94.5 ± 19.2**	**<0.01**
Total cholesterol (mg/dl)	202.6 ± 36.8	193.6 ± 26.0	0.23	**198.5 ± 33.8**	**190.4 ± 29.6**	**0.03**	**198.6 ± 38.5**	**194.0 ± 39.1**	**0.04**	**201.9 ± 40.4**	**190.2 ± 34.9**	**<0.01**
HDL cholesterol (mg/dl)	70.3 ± 15.7	70.0 ± 18.8	0.91	68.9 ± 19.1	66.1 ± 16.9	0.16	69.2 ± 18.9	69.1 ± 18.2	0.94	68.6 ± 15.7	66.6 ± 15.2	0.05
LDL cholesterol (mg/dl)	119.4 ± 28.9	108.6 ± 19.7	0.10	113.6 ± 34.7	111.6 ± 29.4	0.58	**117.5 ± 35.9**	**111.5 ± 35.1**	**<0.01**	**120.4 ± 36.9**	**113.5 ± 34.7**	**<0.01**
Triglycerides (mg/dl)	83.6 ± 35.0	89.1 ± 43.4	0.62	116.6 ± 127.3	98.6 ± 44.9	0.28	103.6 ± 58.8	109.5 ± 62.7	0.10	101.3 ± 50.5	95.3 ± 46.2	0.14
IGF-I (ng/ml)	184.6 ± 71.3	184.8 ± 73.9	0.99	175.3 ± 58.7	164.8 ± 66.3	0.10	170.3 ± 70.0	169.4 ± 62.6	0.80	**179.5 ± 70.8**	**170.3 ± 75.1**	**<0.01**
Insulin (µIU/ml)	19.4 ± 9.6	16.6 ± 13.8	0.53	16.9 ± 10.6	13.6 ± 10.3	0.14	**19.5 ± 17.2**	**14.6 ± 12.5**	**<0.01**	**22.1 ± 20.0**	**13.3 ± 12.0**	**<0.01**
CRP (mg/L)	2.4 ± 1.7	1.6 ± 1.2	0.01	1.6 ± 1.7	2.1 ± 2.8	0.30	2.0 ± 2.7	1.7 ± 2.3	0.18	**1.7 ± 2.1**	**1.4 ± 1.9**	**0.04**

* p of *t*-test for difference between baseline and 6 months by randomization group.

Table 3 reports the results of the intention-to-treat analysis (IG vs. CG) by pathogenic variant type. The “delta” analysis of the differences between the two randomized groups controlling for the center, age, BMI at baseline, and baseline value of the variable under study showed that among LOF variant carriers, there was a significantly more pronounced decrease in the IG compared to the CG of the following parameters: weight (*p* < 0.001); BMI (*p* < 0.001); waist and hip circumferences (*p* = 0.01 and *p* < 0.01, respectively); and the levels of total cholesterol (*p* = 0.04), triglycerides (*p* = 0.02), and insulin (*p* = 0.04) (Table 3). Conversely, in the carriers of nonsynonymous variants, no significant differences were detected for any of the parameters considered, when comparing the changes between the baseline and end-of-treatment values in the IG vs. the CG. Performing the “delta” analysis of differences including the pathogenic variant type into the model, we observed that the difference in insulin level reduction between IG and CG was significantly higher in LOF (−3.9 µIU/ml) compared to nonsynonymous (−0.5 µIU/ml) variant carriers (*p* < 0.01). In the “delta” analysis of differences, a further significant result was obtained concerning the systolic pressure. In the LOF variant carriers, the decrease of the values of this parameter in the IG and CG was virtually equivalent (Δ = −0.1 mmHg). In nonsynonymous variant carriers, the values of systolic pressure remained almost unaltered in IG, while we observed an increase in CG (Δ = −3.1 mmHg; *p* < 0.01). Table 4 reports the changes in the frequencies of food consumption from the 24 h food frequency diaries (intention-to-treat analysis, IG vs. CG) by the pathogenic variant type. The “delta” analysis of differences between the two randomized groups showed that in the IG of both nonsynonymous and LOF variant carriers, there was a significantly higher reduction of the consumption of dairy products compared to the corresponding CG (*p* = 0.04). In addition, the IG of the LOF variant carriers showed a borderline significantly higher reduction of the consumption of red/processed meat and total animal products, and a borderline significantly higher increase of the consumption of whole grains compared to the CG. Including the pathogenic variant type into the model, we did not observe any significant difference among the dietary changes in nonsynonymous variant carriers compared to LOF variant carriers.

**TABLE 3:** Results of intention-to-treat analysis by pathogenic variant type.

	Nonsynonymous CG (*N* = 16)	Nonsynonymous IG (*N* = 38)	p*	LOF CG (*N* = 157)	LOF IG (*N* = 155)	p*	p**
	Δ of differences	Δ of differences		Δ of differences	Δ of differences		
Weight (kg)	−0.7 ± 3.3	−1.4 ± 3.7	0.60	**−0.7 ± 2.4**	**−1.5 ± 2.7**	**<0.01**	0.62
BMI (kg/m^2^)	−0.3 ± 1.3	−0.6 ± 1.4	0.59	**−0.3 ± 0.9**	**−0.6 ± 1.1**	**<0.01**	0.67
Waist circumference (cm)	−0.3 ± 3.1	−2.0 ± 5.8	0.51	**−0.8 ± 4.7**	**−2.1 ± 5.2**	**0.01**	0.54
Hip circumference (cm)	−0.4 ± 2.6	−1.2 ± 3.9	0.50	**−0.5 ± 4.7**	**−1.8 ± 4.3**	**<0.01**	0.36
Systolic pressure (mmHg)	+3.4 ± 11.8	+0.3 ± 16.4	0.94	−4.0 ± 13.4	−4.1 ± 14.9	0.62	**<0.01**
Diastolic pressure (mmHg)	+0.2 ± 6.6	−1.4 ± 10.9	0.78	−2.6 ± 9.8	−2.7 ± 11.1	0.77	0.46
Glycemia (mg/dl)	−10.8 ± 30.5	−8.2 ± 21.8	0.98	−8.8 ± 22.8	−7.3 ± 19.0	0.62	0.64
Total cholesterol (mg/dl)	−9.0 ± 29.0	−8.0 ± 21.6	0.69	**−4.6 ± 28.3**	**−11.7 ± 25.4**	**0.04**	0.84
HDL cholesterol (mg/dl)	+0.3 ± 11.7	+2.8 ± 12.1	0.65	+0.1 ± 13.9	+2.0 ± 12.3	0.09	0.59
LDL cholesterol (mg/dl)	−10.8 ± 24.4	−2.0 ± 22.1	0.22	−6.0 ± 26.1	−6.9 ± 24.5	0.90	0.71
Triglycerides (mg/dl)	+5.5 ± 44.3	−18.0 ± 100.5	0.41	**+5.8 ± 43.6**	**−6.0 ± 49.8**	**0.02**	0.18
IGF-I (ng/ml)	+0.2 ± 39.5	−10.5 ± 38.5	0.42	−0.9 ± 43.2	−9.3 ± 43.1	0.18	0.74
Insulin (µIU/ml)	−2.8 ± 17.8	−3.3 ± 13.2	0.43	**−4.9 ± 18.5**	**−8.8 ± 16.6**	**0.04**	<0.01
CRP (mg/L)	−0.8 ± 1.1	+0.4 ± 2.5	0.29	−0.3 ± 2.5	−0.3 ± 1.7	0.96	0.20

* p of comparison between the randomization groups: ANOVA, controlling for age (tertiles), study center, BMI at baseline (tertiles), and the baseline value of the variable under study.

** p of comparison between the pathogenic variants: ANOVA, including the pathogenic variant type into the model.

**TABLE 4:** Changes in frequencies of food consumption from the 24-h food frequency diaries by pathogenic variant type (intention-to-treat analysis).

	Nonsynonymous CG (*N* = 16)	Nonsynonymous IG (*N* = 38)	p*	LOF CG (*N* = 157)	LOF IG (*N* = 155)	p*	p**
Previous day’s food consumption (times a day)	Δ of differences	Δ of differences		Δ of differences	Δ of differences		
Δ Sugary food and beverages	+0.1 ± 0.9	−0.6 ± 1.0	0.09	−0.4 ± 1.2	−0.3 ± 1.1	0.75	0.99
Δ Refined grain products	−0.4 ± 1.5	−0.6 ± 1.8	0.95	−0.4 ± 1.3	−0.6 ± 1.7	0.16	0.83
Δ Whole-grain products	−0.1 ± 1.4	+0.4 ± 1.4	0.27	+0.1 ± 1.4	+0.4 ± 1.4	0.07	0.65
Δ Legumes and soy products	−0.1 ± 0.6	+0.2 ± 1.1	0.55	−0.1 ± 0.6	+0.1 ± 0.8	0.13	0.74
Δ Dairy products	**−0.1 ± 0.9**	**−0.8 ± 1.1**	**0.04**	**−0.4 ± 0.9**	**−0.6 ± 0.1**	**0.04**	0.91
Δ Red/processed meat	−0.2 ± 0.7	−0.3 ± 0.9	0.98	−0.1 ± 0.7	−0.2 ± 0.8	0.06	0.60
Δ Total animal products	−0.1 ± 1.4	−0.7 ± 2.3	0.64	−0.3 ± 1.3	−0.6 ± 1.9	0.05	0.82
Δ Fish	−0.1 ± 0.9	+0.2 ± 0.9	0.31	−0.1 ± 0.8	+0.1 ± 0.8	0.26	0.51
Δ Vegetables	+0.3 ± 1.7	−0.4 ± 2.8	0.43	−0.1 ± 2.0	+0.3 ± 2.1	0.23	0.30
Δ Fruits	−0.1 ± 1.1	−0.2 ± 1.5	0.99	−0.1 ± 1.2	−0.1 ± 1.5	0.65	0.56
Δ Alcoholic beverages	−0.1 ± 0.6	−0.1 ± 0.5	0.63	−0.1 ± 0.6	0.0 ± 0.5	0.72	0.56
Δ Nuts and seeds	−0.3 ± 0.9	0.0 ± 0.9	0.20	+0.1 ± 0.2	+0.2 ± 0.8	0.11	0.05

* p of comparison between the randomization groups: ANOVA, controlling for age (tertiles), study center and BMI, at baseline (tertiles).

** p of comparison between the pathogenic variants: ANOVA, including the pathogenic variant type into the model.

## Discussion

We had previously shown that in the whole population of *BRCA1/2* mutation carriers who joined our 6-month randomized controlled trial, the dietary intervention based on the Mediterranean diet with moderate protein restriction was effective in reducing IGF-I, body weight, and MS markers but not insulin (Bruno et al., 2020). In the present study, we evaluated in the 366 female *BRCA1/2* mutation carriers from the same trial whether the changes in the levels of metabolic and hormonal factors, in response to the dietary intervention, were influenced by the type of pathogenic variants (LOF vs. nonsynonymous). Our findings indicated at the end of the intervention period that the serum levels of insulin in LOF variant carriers were significantly more reduced than in nonsynonymous variant carriers. These observations support the hypothesis that the LOF of BRCA proteins might result in a different response of metabolic factors to diet, possibly due to a genetic effect. This hypothesis is further supported by the lack of significant differences in the changes of dietary habits when comparing the IG of LOF vs. nonsynonymous variant carriers. It has to be remarked that since we had previously observed that higher levels of insulin at baseline were significantly associated with BRCA LOF variants (Oliverio et al., 2020), the intention-to-treat analysis was carried out controlling for the baseline insulin levels, to rule out a possible bias due to this factor.

Insulin, stimulating mitosis and inhibiting apoptosis, promotes cancer growth (Draznin, 2010). Since BRCA mutations affect the mechanisms of DNA damage repair, the carriers of BRCA mutations may be more sensitive to the mitogenic effect of insulin. Previous studies suggested that nonsynonymous variants in BRCA genes are associated with a reduced risk of BC in comparison to LOF variants (Spurdle et al., 2014; Shimelis et al., 2017; Moghadasi et al., 2018; Li et al., 2021). Furthermore, insulin resistance and obesity are associated with hereditary BC (Bordeleau et al., 2011; Dumais et al., 2017). We found that these conditions are more frequent in *BRCA1/2* LOF carriers. These associations seemed stronger in *BRCA2* carriers (Oliverio et al., 2020). Therefore, the results of the present study, although preliminary, open new perspectives for the investigations of the correlation between metabolic factors and BRCA-related cancer and suggest the possibility to introduce “personalized” dietary treatments in high-risk BRCA variant carriers, as a risk-reduction measure, taking into account the mutation type.

The major limitation of this study is the relatively small number of nonsynonymous variant carriers in our trial population. Therefore, additional similar studies, including a larger number of BRCA mutation carriers, are needed to support our conclusions. Furthermore, without a prospective evaluation, we could not quantify the effects on the BRCA-related cancer risk of the greater reduction of serum insulin observed in LOF variant carriers. To date, no other studies have evaluated the roles of a dietary intervention on BRCA-related cancer in relation to the variant type. Thus, the follow-up of our cohort could provide an evidence-based rationale in support of a healthy lifestyle as a personalized risk-reduction measure for BRCA mutation carriers.

## Data Availability

The original contributions presented in the study are included in the article/Supplementary Materials, further inquiries can be directed to the corresponding author.
