# Supplementary material for: The Impact of Mediterranean Dietary Intervention on Metabolic and Hormonal Parameters According to BRCA1/2 Variant Type
Source: Front Genet. 2022 Mar 9;13:820878. doi: 10.3389/fgene.2022.820878 (PMC8959623; doi:10.3389/fgene.2022.820878)
Supplement: Supplementary file 1 [file Table2.docx]

| **Total Population**  **(N= 366)** | **Control group**  **(N=173)** | | | **Intervention group**  **(N= 193)** | | |
| --- | --- | --- | --- | --- | --- | --- |
|  | **Baseline**  **Mean** ± **SD** | **Six Months**  **Mean** ± **SD** | **p*** | **Baseline**  **Mean** ± **SD** | **Six Months**  **Mean** ± **SD** | **p*** |
| **Weight (Kg)** | **65.4 ± 13.7** | **64.7 ± 13.5** | **< 0.01** | **62.1 ± 10.6** | **60.7 ± 10.7** | **< 0.01** |
| **BMI (kg/m²)** | **24.7 ± 4.9** | **24.5 ± 4.8** | **< 0.01** | **23.9 ± 4.4** | **23.3 ± 4.3** | **< 0.01** |
| **Waist circumference (cm)** | **78.9 ± 13.2** | **78.2 ± 12.5** | **0.03** | **77.0 ± 10.5** | **75.1 ± 10.4** | **< 0.01** |
| **Hip circumference (cm)** | 100.8 ± 10.1 | 100.3 ± 10.2 | 0.14 | **98.4 ± 9.1** | **96.8 ± 8.8** | **< 0.01** |
| **Systolic pressure (mmHg)** | **124.4 ± 15.5** | **121.0 ± 13.8** | **< 0.01** | **126.1 ± 18.0** | **122.9 ± 14.8** | **< 0.01** |
| **Diastolic pressure (mmHg)** | **81.3 ± 10.6** | **79.0 ± 9.2** | **< 0.01** | **82.3 ± 11.2** | **79.9 ± 10.9** | **< 0.01** |
| **Glycemia (mg/dL)** | **102.1± 24.2** | **93.1 ± 20.1** | **< 0.01** | **101.1 ± 22.2** | **93.6 ± 18.4** | **< 0.01** |
| **Total cholesterol (mg/dL)** | **199.0 ± 38.3** | **194.0 ± 38.0** | **0.02** | **201.2 ± 39.1** | **190.3 ± 33.8** | **< 0.01** |
| **HDL cholesterol (mg/dL)** | 69.3 ± 18.6 | 69.2 ± 18.2 | 0.92 | **68.7 ± 16.4** | **66.5 ± 15.5** | **0.02** |
| **LDL cholesterol (mg/dL)** | **117.7 ± 35.2** | **111.2 ± 34.0** | **< 0.01** | **119.0 ± 36.5** | **113.1 ± 33.7** | **< 0.01** |
| **Triglycerides (mg/dL)** | 101.8 ± 57.2 | 107.6 ± 61.4 | 0.08 | 104.3 ± 72.2 | 96.0 ± 45.9 | 0.07 |
| **IGF-I (ng/mL)** | 171.6 ± 61.9 | 170.8 ± 63.6 | 0.81 | **178.7 ± 68.5** | **169.2 ± 73.3** | **< 0.01** |
| **Insulin (µIU/ml)** | **19.5 ± 16.7** | **14.8 ± 12.6** | **< 0.01** | **21.1 ± 18.6** | **13.4 ± 11.6** | **< 0.01** |
| **CRP (mg/L)** | 2.0 ± 2.6 | 1.7 ± 2.6 | 0.08 | 1.7 ± 2.0 | 1.5 ± 2.2 | 0.29 |

**Supplementary Table 2.** Results of before-after analysis by randomization group

* p of t-test for difference between baseline and six months
